# Clinical Outcomes after Intravenous Alteplase in Elderly Patients with Acute Ischaemic Stroke: A Retrospective Analysis of Patients Treated at a Tertiary Neurology Centre in England from 2013 to 2018

**DOI:** 10.1155/2021/3738017

**Published:** 2021-10-31

**Authors:** Xuya Huang, Phillip Nash, Vafa Alakbarzade, Brian Clarke, Anthony C. Pereira

**Affiliations:** ^1^Neurology Department, Institute of Neurological Sciences, Queen Elizabeth University Hospital, 1345 Govan Road, Glasgow G514TF, UK; ^2^Neurology Department, St George's University Hospitals NHS Foundation Trust, Backshaw Road, London, SW170QT, UK

## Abstract

Intravenous thrombolysis with alteplase within 4.5 hours from symptom onset is a well-established treatment of acute ischaemic stroke (AIS). The aim was to compare alteplase for AIS between patients aged >80 and ≤80 years in our registry data, from 2013 to 2018. Mechanical thrombectomy cases were excluded. We assessed clinical outcomes over the six-year period and between patients aged over 80 and ≤80 years, using measures including the discharge modified Rankin Scale (mRS), 24-hour National Institutes of Health Stroke Scale (NIHSS) improvement, and symptomatic intracerebral haemorrhage (sICH) rate. Of a total of 805 AIS patients who received intravenous alteplase, 278 (34.5%) were over 80 years old, and 527 (65%) were younger. 616 (76.5%) received thrombolysis ≤ 3 hours after symptom onset and 189 (23.5%) within 3-4.5 hours. Median baseline mRS and NIHSS of the elderly cohort were 1 (IQR 0-5) and 13 (IQR 2-37), respectively, compared to the younger cohort 0 (IQR 0-5) and 9 (IQR 0-29). The sICH rate was 7.2% in the elderly and 4.6% in those ≤80 years, *p* = 0.05. NIHSS improved within 24 hours in 34% of the elderly cohort compared to 35% in the younger cohort. At hospital discharge, the mortality rate was 9% in the elderly cohort compared to the 6% in the younger cohort, *p* = 0.154. 25% of patients aged >80 years had mRS ≤ 2 compared to 47% in the younger patients (*p* < 0.0001). In conclusion, thrombolysis in elderly patients results in clinical improvement comparable to younger patients.

## 1. Introduction

Intravenous (IV) thrombolysis is a well-established treatment for AIS. Its use has been widespread for stroke within three hours of onset since the National Institute for Neurological Disorders (NINDS) trial reported in 1995 [1]. Thrombolysis for AIS up to 4.5 hours after onset became a common practice after the results of the European Cooperative Acute Stroke Study (ECASS) III trial were published in 2008 [2]. However, this study had stringent criteria, excluding patients aged over 80 years and those with a combination of previous stroke and diabetes mellitus. Subsequently, evidence emerged that treating patients over 80 was appropriate [3, 4] with the most recently updated meta-analysis [5] including all IV thrombolysis trials comparing alteplase with placebo, confirming that the elderly population benefitted from treatment. Now, IV thrombolysis is the standard hyperacute reperfusion therapy for AIS within 4.5 hours from symptom onset in all adult age groups worldwide.

At our centre, we have performed thrombolysis for stroke since 2004. In 2010, stroke care in London was reorganised to create eight Hyperacute Stroke Units (HASUs) where the majority of stroke cases are first admitted. One of the main objectives of this was to facilitate the delivery of urgent reperfusion therapy. The HASU model was very successful with an increase in thrombolysis rates from 5% to 13% and decrease in mortality of 3% [6, 7]. In 2013, the Sentinel Stroke National Audit Programme (SSNAP) was started, which replaced previous sentinel audits of stroke. We have been entering data into SSNAP since its inception. It provides a readily accessible method for identifying stroke patients and analysing data to audit a unit's performance. Here, we investigated the elderly compared with the younger stroke patients' clinical outcomes after IV thrombolysis.

## 2. Materials and Methods

This is a retrospective study of our data (from a tertiary neurology center in England) entered prospectively in the UK SSNAP audit identifying all patients thrombolysed between January 2013 and December 2018. SSNAP has permission from the NHS Health Research Authority under section 251 of the Health and Social Care Act 2006 to collect patient data without prospective consent. We defined the outcomes as favorable clinical outcome (modified Rankin Scale (mRS) ≤ 1), independence in activities of daily living (mRS ≤ 2) at hospital discharge, neurological improvement at 24 hours after IV alteplase (National Institutes of Health Stroke Scale (NIHSS) improvement ≥ 8 or score = 0-1), rate of symptomatic intracranial haemorrhage (sICH), and mortality rate. We excluded patients if (1) they underwent mechanical thrombectomy and (2) symptom onset to treatment was >4.5 hours.

Data were compared using chi-squared tests or Mann-Whitney *U* tests. Multiple logistic regression models were used to assess the association between age groups and binary clinical outcomes and adjusted for patient characteristics (comorbidities, prestroke mRS score, and NIHSS score). The shift of clinical outcomes was compared using ordinal regression analysis. All statistical tests were two-sided, and *p* values < 0.05 were considered to be statistically significant. Statistical analysis was performed in SPSS (V.22; SPSS Inc, Chicago, Illinois, USA). No external funding was involved in the conduct of this study.

## 3. Results

Over six years, 805 patients with acute ischaemic stroke who received alteplase were identified. 278 (34.5%) patients were over 80 years of age (26% were 80–89 years and 8% ≥ 90 years) versus 527 (65%) in the younger cohort. 616 (76.5%) received alteplase within 3 hours after symptom onset versus 189 (23.5%) within 3-4.5 hours after symptom onset. Baseline characteristics are shown in Table 1. Median baseline mRS and NIHSS of the elderly cohort were 1 (IQR 0-5) and 13 (IQR 2-37), respectively, compared to the younger cohort (0 (IQR 0-5) and 9 (IQR 0-29)). The median number of comorbidities was similar in both subgroups (1; IQR 0-2). There was no significant difference in the rates of atrial fibrillation (AF), hypertension, previous stroke, or TIA between the ≤80 and the over 80 groups (Table 1).

At hospital discharge, mortality was 9% (25/278) in the older cohort compared to 6% (33/527) in the younger cohort, *p* = 0.154. Considering the 0–3 vs. 3–4.5-hour time-to-treatment windows, mortality at hospital discharge in the older group was 10% (22/212) and 4% (3/66), respectively (*p* = 0.12). The sICH rate was 5.5% (44/805) overall. In the older cohort, it was 7.2% (20/278) compared to 4.6% (24/527) in the younger (*p* = 0.05). In the 3-hour time-to-treatment window, sICH rates were 7.5% in the older cohort compared to 4.5% in the younger, *p* = 0.11. In the 3-4.5-hour treatment window, it was 6% compared to 4.9% in the younger (*p* = 0.76) (Table 2).

24 hours after alteplase, the NIHSS score worsened in 12% (33/278) of the older cohort compared to 14% (71/524) in the younger (*p* = 0.5). Neurological improvement occurred in 34% (94/278) of the older cohort compared to 35% (186/527) of the younger. 38 (13.7%) patients over 80 years had mRS ≤ 1 post thrombolysis by the time of discharge compared to 186 (35%) of the younger group (*p* < 0.0001). 25% (69/278) of patients aged over 80 years had mRS ≤ 2 compared to 47% (248/527) of younger patients (*p* < 0.0001) at discharge. Alteplase administration within 3 hours of symptom onset reduced mRS to ≤2 at hospital discharge in 54/212 (25.5%) of the older cohort compared to 186/404 (46.0%) in the younger, *p* < 0.0001. Alteplase administration 3–4.5 hours after onset in the older cohort reduced mRS to ≤2 at hospital discharge in 15/66 (22.7%) of the older cohort compared to 62/123 (50.4%) in the younger, *p* = 0.0001 (Table 2). Over the 6-year period, the proportion of patients achieving a good functional outcome increased (Supplementary Figure 1), with a relatively stable proportion of patients suffering severe disability or mortality (mRS 5-6).

In multiple logistic regression analysis, independence at hospital discharge was associated with younger age (OR (95% CI), 0.08 (0.01-0.15), *p* = 0.02), better prestroke mRS (OR (95% CI), 0.07 (0.04-0.09), *p* < 0.0001), and lower admission NIHSS score (OR (95% CI), 0.02 (0.019-0.028), *p* < 0.0001) after adjustment for prior stroke event, diabetes, AF, hypertension, congestive heart failure, and time-to-treatment windows (Table 3).

**Figure 1:**
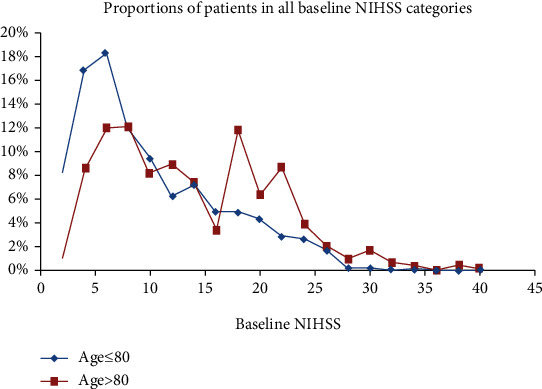
The comparison of proportions of patients in all stroke severity in two age groups. NIHSS: National Institutes of Health Stroke Scale. The area under each curve adds up to 100%.

Figure 1 demonstrates that in the younger age group, the majority of patients had milder (NIHSS ≤ 10) stroke syndromes. There was a bigger proportion of patients with very mild stroke syndromes (NIHSS ≤ 5) in the younger group compared with the older group (33.8% vs. 14.4%, *p* < 0.001). Interestingly, a significantly higher proportion of older patients who presented with substantial stroke syndromes (NIHSS ≥ 16) were thrombolysed (39.6% vs. 19.7%, *p* < 0.001).

## 4. Discussion

In our retrospective, single-centre, large cohort analysis, we observed a higher rate of alteplase administration to elderly stroke patients compared to the national average [8] (34.5% vs. 11%), pooled cohort of randomized trials, and European SITS-MOST registry database (28.6%) [9, 10]. Stroke tends to disproportionately affect older individuals [11], so data on the risks and benefits of alteplase in the older population is important. We explored the safety profile of alteplase in >80-year-old cohort and their outcome at hospital discharge compared to younger patients.

Table 2 shows that in either time-to-treatment window, the older cohort had a much lower chance of enjoying an excellent outcome or independence at discharge; this was highly significant. Table 1 provides one possible explanation for this. It shows that at baseline, the older patients in our cohort on average had a worse baseline clinical status (higher mRS) and presented with larger syndromes (measured by NIHSS). Overall, the outcome in the elderly group was poorer; mortality at hospital discharge in our treated elderly cohort was comparable to those under 80 years of age (*p* = 0.154). Our results agree with the Third International Stroke Trial (IST-3) that showed no overall difference in long-term mortality in the elderly patients who received alteplase versus standard care alone [12, 13]. Furthermore, the sICH rate in our overall cohort was comparable to that in published data [5] and in our elderly cohort specifically, while higher than that in the younger group (7.2% vs. 4.6%) but did not quite reach statistical significance (*p* = 0.05). This is consistent with a recent registry study [9] that showed that the incidence of sICH and overall mortality following thrombolysis of patients aged >80 years was not increased in routine practice versus clinical trials [12, 13]. Time-to-treatment windows (0–3 vs. 3–4.5 hours) did not have an impact on case fatality (*p* = 0.12) or sICH rate (*p* = 0.47) in our elderly cohort.

However, our data does provide a novel insight: the proportion of patients who improved within 24 hours of treatment was similar irrespective of age. In fact, a greater proportion of the older group improved in the later time window than their younger counterparts (Table 2). This implies that there was a group of patients who were able to benefit from treatment irrespective of their age, severity of syndrome, or preexisting disability. This supports evidence [12] that alteplase is as effective in older as in younger patients compared to placebo. We speculate that some of these patients may have a core/penumbra mismatch which manifests as substantial early clinical improvement following successful thrombolysis. Such core/penumbra mismatch has been demonstrated with perfusion imaging [14, 15] and has been utilised successfully in clinical trials [16, 17].

Pooled analysis of randomized trials and the European SITS-MOST registry [9, 10] showed better outcomes in younger versus older patients in both time-to-treatment periods. Interestingly, elderly patients with severe stroke were excluded from both these studies [9, 10], as were those with history of diabetes mellitus and stroke. Compared to these studies, more elderly patients in our centre received alteplase within 3–4.5 hours of onset of symptoms (35% vs. 22%) [9, 10]. Our elderly cohort also included 5% of those with history of diabetes mellitus and stroke (13%). Thus, our analysis had sufficient power to inform the relationship between age, early neurological improvement, and treatment time beyond three hours. Early neurological improvement is reported to be the best predictor or surrogate marker of 3-month functional outcome and recanalization after thrombolysis [18–20]. Therefore, our study provides support for thrombolysis in the elderly.

Advanced stroke imaging may help inform which patients aged over 80 should receive IV thrombolysis based on individual assessment, such as Computer Tomography (CT) perfusion [21–23]. We suggest that the routine use of advanced imaging in stroke thrombolysis may better focus its administration.

Our data intriguingly also showed that significantly more elderly patients with big stroke syndromes (about NIHSS 16) were thrombolysed within three hours (Figure 1). The reason for this is not evident, but we speculate that the treating physician, patient, and their family may have felt that there was little to lose in accepting treatment. This observation may explain why our elderly cohort was treated in spite of having more severe stroke syndromes than their younger counterparts.

Several limitations of our study have to be noted including methodological limitations related to the retrospective analysis. We did not have three-month outcome data available. Therefore, our results appear not to tally with IST-3 findings [12], but this is probably because our outcome was “discharge mRS” while the outcome in IST-3 was “3-month mRS.” Patients would have received more rehabilitation and made further improvement by 3 months post stroke. However, our well-kept stroke registry (with minimal missing data points and consecutive patients) did not identify a selection bias favoring the younger cohort. There was no evidence that younger, less disabled patients were selectively chosen for thrombectomy and therefore excluded from this study, while older, more disabled patients were treated with alteplase alone.

## 5. Conclusions

Over a 6-year period, our ability to deliver thrombolysis with alteplase safely in a very large elderly cohort adds additional reassurance that alteplase does not cause more harm in patients aged >80 years than in younger adults. Older patients had worse outcomes, probably because they had larger stroke syndromes and were more disabled at baseline. However, the early treatment response was similar in both groups implying that treatment was justified. We believe that further studies could help identify patients more likely to respond to better focussed treatment. Routine use of perfusion imaging (to better select eligible patients for thrombolysis) may enhance thrombolysis decisions and outcomes in elderly patients in the future.

## Figures and Tables

**Table 1:** Basic characteristics between the groups of aged >80 and aged ≤80 according to treatment window.

Characteristics	Time to treatment 0–3 h *N* = 616			Time to treatment 3–4.5 h *N* = 189		
	Age ≤ 80 *N* = 404	Age > 80 *N* = 212	*p* value	Age ≤ 80 *N* = 123	Age > 80 *N* = 66	*p* value
Male, No. (%)	262 (64.9%)	83 (39.2%)	<0.001	77 (62.6%)	30 (45.5%)	0.03
Baseline NIHSS, median (IQR)	8 (5-14)	12 (7-19)	<0.001	7 (4-12)	11.5 (7-17)	0.005
Baseline mRS, median (IQR)	0 (0-0)	1 (0-2)	<0.001	0 (0-0)	1 (0-2)	<0.001
Hypertension, No. (%)	259 (64.1%)	131 (61.8%)	0.6	76 (61.8%)	41 (62.1%)	1
Atrial fibrillation^∗^, No. (%)	88 (21.8%)	46 (21.7%)	1	28 (22.8%)	8 (12.1%)	0.083
History of stroke/TIA, No. (%)	87 (21.5%)	41 (19.3%)	0.6	25 (20.3%)	13 (19.7%)	1
Congestive heart failure, No. (%)	35 (8.7%)	14 (6.6%)	0.44	8 (6.5%)	3 (4.5%)	0.75
Diabetes mellitus, No. (%)	93 (23%)	49 (23.1%)	1	30 (24.4%)	8 (12.1%)	0.057
Number of comorbidities, median (IQR)	1 (0-2)	1 (0-2)	0.42	1 (0-2)	1 (0-1)	0.068

^∗^Atrial fibrillation diagnosed from an admission 12-lead ECG; IQR: interquartile range; mRS: modified Rankin Scale; NIHSS: National Institutes of Health Stroke Scale; No.: number; TIA: transient ischaemic attack.

**Table 2:** Clinical and safety outcomes in the groups of aged >80 and aged ≤80 according to treatment window.

Outcomes	Time to treatment 0–3 h *N* = 616			Time to treatment 3–4.5 h *N* = 189		
	Age ≤ 80 *N* = 404	Age > 80 *N* = 212	*p* value	Age ≤ 80 *N* = 123	Age > 80 *N* = 66	*p* value
Mortality rate^∗^, No. (%)	26 (6%)	22 (10%)	0.08	7 (6%)	3 (4%)	0.7
Symptomatic ICH rate, No. (%)	18 (4.5%)	16 (7.5%)	0.11	6 (4.9%)	4 (6%)	0.76
Favorable outcome^∗∗^, No. (%)	136 (34%)	33 (16%)	<0.0001	50 (41%)	5 (7%)	<0.0001
Independence^∗∗∗^, No. (%)	186 (46%)	54 (26%)	<0.0001	62 (50%)	15 (22%)	0.0001
Neurological improvement^∗∗∗∗^, No. (%)	152 (41.8%)	73 (37.4%)	0.37	34 (31.8%)	21 (35.6%)	0.73

^∗^At hospital discharge; ^∗∗^modified Rankin Scale ≤ 1 at hospital discharge; ^∗∗∗^modified Rankin Scale ≤ 2 at hospital discharge; ^∗∗∗∗^baseline National Institutes of Health Stroke Scale score improvement ≥ 8 or remained at ≤1 24 hours after intravenous thrombolysis; No.: number; ICH: intracerebral haemorrhage.

**Table 3:** Independence at hospital discharge.

Characteristics	Age ≤ 80 *N* = 527	Age > 80 *N* = 278	Odds ratio (95% CI)	*p* value
Independence^∗^, No. (%)	248 (47%)	69 (25%)	0.08 (0.01-0.15)	0.02
Baseline NIHSS score, median (IQR)	9 (0-29)	13 (2-37)	0.02 (0.019-0.028)	<0.0001
Baseline mRS, median (IQR)	0 (0-5)	1 (0-5)	0.07 (0.04-0.09)	<0.0001
Hypertension, No. (%)	335 (64%)	172 (62%)	0.04 (-0.74-0.26)	0.70
Atrial fibrillation^∗∗^, No. (%)	116 (22%)	54 (19%)	0.63 (-0.30-0.42)	0.73
History of stroke/TIA, No. (%)	112 (21%)	54 (19%)	0.18 (-0.18-0.22)	0.86
Congestive heart failure, No. (%)	43 (8%)	17 (6%)	0.06 (-0.16-0.29)	0.58
Diabetes mellitus, No. (%)	94 (18%)	57 (21%)	0.23 (-0.09-0.56)	0.16

^∗^Modified Rankin Scale ≤ 2 at hospital discharge; ^∗∗^atrial fibrillation diagnosed from an admission 12-lead ECG; IQR: interquartile range; mRS: modified Rankin Scale; NIHSS: National Institutes of Health Stroke Scale; No.: number; TIA: transient ischaemic attack.

## Data Availability

Data availability is on request.

## Abbreviations

AF: Atrial fibrillation
AIS: Acute ischaemic stroke
CT: Computer Tomography
ECASS: European Cooperative Acute Stroke Study
HASU: Hyperacute Stroke Units
IQR: Interquartile range
IST-3: International Stroke Trial
IV: Intravenous
mRS: Modified Rankin Scale
NIHSS: National Institute of Health Stroke Scale
NINDS: National Institute for Neurological Disorders
PRACTISE: Penumbra and Recanalisation Acute Computed Tomography in Ischaemic Stroke Evaluation
sICH: Symptomatic intracerebral haemorrhage
SSNAP: Sentinel Stroke National Audit Programme
TIA: Transient ischaemic attack.

## Supplementary Materials

Supplementary Materials Supplementary Figure 1: the comparison of proportions of patients in all stroke severity in two age groups. The distribution of discharge modified Rankin Scale, using Friedman's two-way ANOVA test, *p* < 0.001. In the latter two years, thrombectomy numbers picked up, and this probably biased the outcome.
