# Supplementary figures and images for: Clinical Outcomes after Intravenous Alteplase in Elderly Patients with Acute Ischaemic Stroke: A Retrospective Analysis of Patients Treated at a Tertiary Neurology Centre in England from 2013 to 2018
Source: Stroke Res Treat. 2021 Oct 31;2021:3738017. doi: 10.1155/2021/3738017 (PMC8572621; doi:10.1155/2021/3738017)

### Proportion of good vs poor outcomes at discharge over 6 years

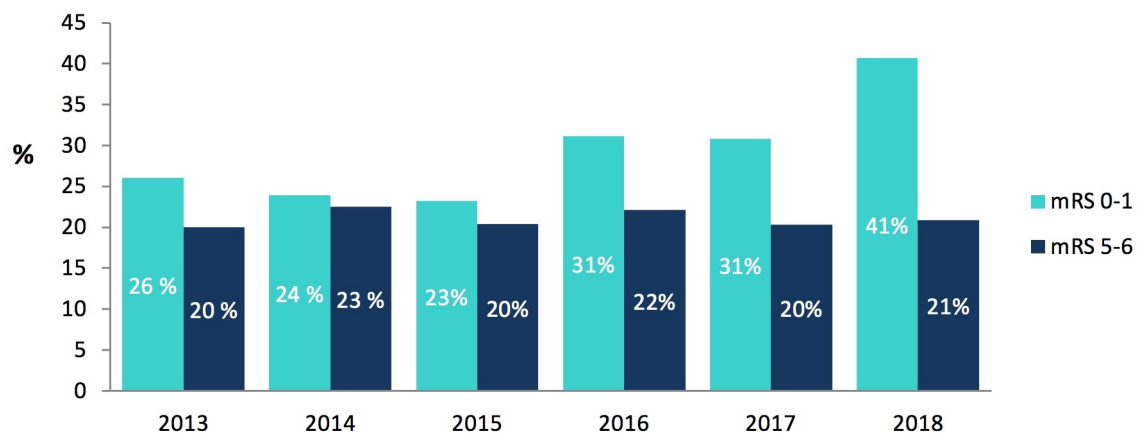

Supplement: Supplementary Materials — Supplementary Figure 1: the comparison of proportions of patients in all stroke severity in two age groups. The distribution of discharge modified Rankin Scale, using Friedman's two-way ANOVA test, p < 0.001. In the latter two years, thrombectomy numbers picked up, and this probably biased the outcome. [file 3738017.f1.pdf]
